# Utility of the Platelet-to-Lymphocyte Ratio in Predicting Advanced Liver Fibrosis in the Hepatitis C Virus (HCV)-Infected Population

**DOI:** 10.7759/cureus.82882

**Published:** 2025-04-24

**Authors:** Vijesh Kumar, Muhammad Aslam, Sanaullah Kalwar, Ali Hyder, Khaild Tareen, Sandeep Kumar, Raja Taha Yaseen Khan, Abbas A Tasneem, Nasir Hassan Luck

**Affiliations:** 1 Hepatogastroenterology, Sindh Institute of Urology and Transplantation, Karachi, PAK; 2 Gastroenterology, Madinah Teaching Hospital, Faislabad, PAK; 3 Gastroenterology and Hepatology, Gambat Institute of Medical Sciences, Gambat, PAK; 4 Gastroenterology, Chandka Medical College, Shaheed Mohtarma Benazir Bhutto Medical University, Larkana, PAK; 5 Gastroenterology, Sheikh Khalifa Bin Zayed Al Nahyan Medical Complex, Quetta, PAK

**Keywords:** aspartate aminotransferase-to-platelet ratio index (apri score), fib-4 index, hcv, liver fibrosis, non-invasive predictors, platelet-to-lymphocyte ratio (plr)

## Abstract

Introduction

Hepatitis C virus (HCV) infection is still a worldwide health issue, leading to progressive liver fibrosis, cirrhosis, and hepatocellular carcinoma. Detection at early stages of advanced liver fibrosis is critical for early treatment and appropriate management. Liver biopsy, though still considered the gold standard for fibrosis staging, is invasive and costly and poses possible risks and complications. The application of non-invasive biomarkers such as the platelet-to-lymphocyte ratio (PLR) as substitute tools for fibrosis staging is on the rise. This study aimed to determine the utility of PLR in predicting advanced liver fibrosis in HCV infection.

Methodology

This retrospective observational study was carried out at the department of hepatogastroenterology, Sindh Institute of Urology and Transplantation (SIUT), Pakistan, in patients aged ≥18 years old who had established chronic infection of HCV and had undergone liver biopsy and shear wave elastography (SWE) in the period between January 2018 and December 2023. Exclusion criteria consisted of coexisting liver and hematological disorders and incomplete patient clinical records. Laboratory parameters, demographic variables, and fibrosis scores were compared. The ratio of PLR was calculated. Area under the receiver operating characteristic (AUROC) curve analysis was done for PLR, and at an optimal cutoff, diagnostic accuracy was obtained for PLR and was compared to aspartate aminotransferase-to-platelet ratio index (APRI) and fibrosis-4 index (FIB-4).

Results

A total of 107 HCV-infected patients, who had a mean age of 49.1 ± 8.9 years, were enrolled in the study. Liver biopsy confirmed significant fibrosis (meta-analysis of histological data in viral hepatitis (METAVIR) score ≥ 2) in 56 (52.3%) patients. The PLR was significantly lower in patients with advanced fibrosis (4.17 ± 1.44 vs. 6.8 ± 1.99, p ≤ 0.001). The AUROC for PLR was 0.879 (p ≤ 0.001). At an optimal cutoff of ≤5.41, PLR showed a high sensitivity of 85.71%, specificity of 86.27%, and an excellent diagnostic accuracy of 85.98%. The diagnostic accuracy of PLR was far superior to APRI (37%) and FIB-4 (40%) in predicting advanced liver fibrosis in HCV patients.

Conclusion

PLR is a simple, cost-effective, highly sensitive, non-invasive marker for advanced liver fibrosis in chronic infection with HCV. It is superior to currently established non-invasive markers such as APRI and FIB-4 and can be utilized as a good screening tool for fibrosis in resource-limited situations.

## Introduction

Hepatitis C virus (HCV) infection is still a global disease affecting millions worldwide [1,2]. Chronically, HCV is one of the most common causes of liver fibrosis, eventually leading to cirrhosis, hepatocellular carcinoma, and liver failure [3,4]. The outcome for HCV patients relies on the early diagnosis of advanced liver fibrosis, allowing for the provision of early treatment with antivirals for the prevention of the progression of liver dysfunction [5].

Traditionally, liver biopsy has been considered the gold standard for fibrosis measurement. Apart from being expensive and its invasiveness, it is also associated with certain limitations, including causing pain at the biopsy site and the potential for certain complications like bleeding [6]. Recently, extensive research has been done on evaluating the role of minimally invasive predictors of liver fibrosis like gamma-glutamyl transferase (GGT) [7], international normalized ratio (INR) to platelet ratio (INPR) [8], aspartate aminotransferase-to-platelet ratio index (APRI), and fibrosis-4 index (FIB-4) [9].

Platelet-to-lymphocyte ratio (PLR) is a novel non-invasive marker used not only for the prediction of infections such as *Helicobacter pylori *infection [10,11]; but also has shown promise regarding its role in predicting progression to cirrhosis in patients with non-alcoholic fatty liver disease (NAFLD) [12]. However, the data is scarce regarding its role in predicting advanced liver fibrosis in HCV infection. Alsebaey et al. has proposed the role of PLR in not only predicting advanced liver fibrosis in the HCV population but also in predicting insulin resistance in this subset of patients [13].

PLR is a simple parameter from complete blood count (CBC) tests [10]. PLR represents the ratio of thrombopoiesis and overall inflammation, both of which participate in fibrosis of the liver. Reduction of the count of platelets has been regularly encountered in patients with progressive fibrosis of the liver, primarily due to splenic sequestration and low levels of thrombopoietin. Lymphocytes, being responsible for immune response, may be modified under chronic inflammation [14]. Thus, a ratio of two blood parameters, PLR, may be regarded as an indirect marker of fibrotic status and of the inflammatory milieu of the liver.

Several studies have compared the predictive value of non-invasive parameters, including the gamma-glutamyl transpeptidase (GPR), APRI, and FIB-4 for liver fibrosis [9]. Although such parameters were relatively accurate, there remains a need for improvement, particularly in environments where access may be limited for expensive imaging modalities and costly serological assays.

Therefore, there is a need for an accurate, non-invasive marker in predicting advanced liver fibrosis that can avoid liver biopsy. The primary goal of this study was to determine the utility of the PLR as a simple, non-invasive predictor of advanced liver fibrosis in patients with chronic HCV infection.

## Materials and methods

Study design

After the approval from the Ethical Review Committee (ERC), Sindh Institute of Urology and Transplantation (SIUT) (approval no.: 1063), this retrospective observational study was conducted at the department of hepatogastroenterology, SIUT, Karachi, Pakistan. All the patients with age greater than 18 years and a confirmed diagnosis of chronic hepatitis C infection attending the outpatient department from January 1, 2018, to December 31, 2023, were included based on their clinical records, availability of laboratory data including CBC, and if they had undergone both shear wave elastography (SWE) and liver biopsy before starting treatment with antiviral therapy. Excluded patients include those with co-existing liver diseases such as hepatitis B, autoimmune hepatitis, alcoholic liver disease, or non-alcoholic liver disease (NAFLD); those patients with concurrent hematological disorders that could affect platelet or lymphocyte counts; those with incomplete clinical records or missing data regarding CBC or fibrosis assessment (SWE and liver biopsy); and those patients receiving treatments that might significantly alter inflammatory or hematological parameters (e.g., immunosuppressive therapy).

Data collection procedure

Data was retrospectively extracted from electronic medical records. Demographic information such as age and gender, along with laboratory parameters including CBC comprising platelet and lymphocyte counts, liver function tests, and findings of SWE and liver biopsy, were recorded. The PLR was calculated for each patient by dividing the absolute platelet count by the absolute lymphocyte count. APRI and FIB-4 were also calculated for each patient.

Data analysis procedure

Statistical analysis was performed using IBM SPSS Statistics for Windows, Version 25 (Released 2017; IBM Corp., Armonk, New York, United States). Continuous variables were expressed as mean ± standard deviation, while categorical variables were expressed as frequencies and percentages. Outcome was observed as the presence or absence of advanced liver fibrosis (meta-analysis of histological data in viral hepatitis (METAVIR) score ≥ F2) on liver biopsy.

Comparative analyses between patients with advanced liver fibrosis and those with less severe disease were conducted using independent sample t-tests for continuous variables and chi-square tests for categorical variables. To evaluate the diagnostic performance of the PLR, receiver operating characteristic (ROC) curve analysis was performed to determine the area under the curve. The optimal cutoff value for PLR was derived using Youden’s index, and the sensitivity, specificity, positive predictive value (PPV), negative predictive value (NPV), and overall diagnostic accuracy were calculated. The diagnostic accuracy of PLR was then compared with APRI (using cutoff ≥1), FIB-4 (using cutoff ≥3.25), and SWE (≥F2) in predicting advanced liver fibrosis in patients with HCV infection.

## Results

A total of 107 HCV-infected patients were enrolled in the study. Mean age was 49.1 ± 8.9 years. Out of 107 patients, 60 (56.1%) patients were males. Using SWE, six (5.6%) patients had F1 fibrosis, 16 (14.9%) had F2 fibrosis, 42 (39.3%) had F3 fibrosis, and 43 (40.2%) had F4 fibrosis. On liver biopsy, 56 (52.3%) patients were found to have significant fibrosis (METAVIR score ≥ 2) (Table 1).

**Table 1:** Baseline characteristics of the studied population SWE: shear wave elastography; TLC: total leukocyte count; ALP: alkaline phosphatase; AST: aspartate transaminase; ALT: alanine transaminase; GGT: gamma-glutamyl transpeptidase; INR: international normalized ratio; PLR: platelet-to-lymphocyte ratio; APRI: aspartate aminotransferase-to-platelet ratio index; FIB-4: fibrosis-4 index Reference ranges: Hb: 12-16 (g/dl); TLC: 4-11 (×10^9^/L); platelet: 150-400 (×10^9^/L); neutrophil count: 15-80 (p/ml); lymphocyte count: 10-48 (p/ml); creatinine: less than or equal to 1.2 mg/dl; serum albumin: 3.6-4 (g/dl); total bilirubin: 0.8-1.0 (mg/dl); ALP: 116-126 (U/L); AST: up to 40 (U/L); ALT: up to 40 (U/L); GGT: up to 50 (U/L); INR: 1.2-1.4

Variables		n (%)
Gender	Male	60 (56.1)
	Female	47 (43.9)
Mean age (years)		49.1 ± 8.9
SWE	F1	6 (5.6)
	F2	16 (14.9)
	F3	42 (39.3)
	F4	43 (40.2)
Liver fibrosis	Yes	56 (52.3)
	No	51 (47.7)
Hemoglobin (g/dl)		11.1 ± 2.5
TLC (×10^9^/L)		7.6 ± 2.6
Platelet (×10^9^/L)		127 ± 52.71
Neutrophil count (p/ml)		63.2 ± 7.9
Lymphocyte (/ml)		28.6 ± 6.7
Serum creatinine (mg/dl)		0.82 ± 0.31
Total bilirubin (mg/dl)		1.1 ± 0.52
ALP (U/L)		168 ± 49
AST (U/L)		45.6 ± 25.5
ALT (U/L)		40.7 ± 18.1
GGT (U/L)		102.9 ± 50.7
Serum albumin (g/dl)		3.2 ± 0.58
INR		1.2 ± 0.24
PLR		5.4 ± 2.14
APRI		0.89 ± 0.6
FIB-4		2.8 ± 1.93

On comparative analysis, patients with advanced liver fibrosis had a lower platelet count (122 ± 33 vs. 177 ± 39) (p ≤ 0.001) as well as a higher INR (1.3 ± 0.27 vs. 1.1 ± 0.19) (p = 0.004) as compared to the patients with no or mild liver fibrosis. The total count of leukocytes (TLC) was also significantly lower in patients with advanced fibrosis (6.8 ± 2.7 vs. 8.3 ± 2.2) (p=0.003). PLR was also significantly lower in patients with advanced fibrosis (4.17 ± 1.44 vs. 6.8 ± 1.99) (p ≤ 0.001), which points toward its usefulness as an excellent predictor of advanced liver fibrosis in patients with HCV infection. APRI as well as FIB-4 were also significantly higher in patients with advanced liver fibrosis (Table 2).

**Table 2:** Comparison of variables in predicting advanced liver fibrosis in patients with HCV (n = 107) TLC: total leukocyte count; ALP: alkaline phosphatase; AST: aspartate transaminase; ALT: alanine transaminase; GGT: gamma-glutamyl transpeptidase; INR: international normalized ratio; PLR: platelet-to-lymphocyte ratio; APRI: aspartate aminotransferase-to-platelet ratio index; FIB-4: fibrosis-4 index Reference ranges: Hb: 12-16 (g/dl); TLC: 4-11 (×10^9^/L); platelet: 150-400 (×10^9^/L); neutrophil count: 15-80 (p/ml); lymphocyte count: 10-48 (p/ml); creatinine: less than or equal to 1.2 mg/dl; serum albumin: 3.6-4 (g/dl); total bilirubin: 0.8-1.0 (mg/dl); ALP: 116-126 (U/L); AST: up to 40 (U/L); ALT: up to 40 (U/L); GGT: up to 50 (U/L); INR: 1.2-1.4

Variables		Advanced liver fibrosis		p-value
		Yes (n = 56), n (%)	No (n = 51), n (%)	
Gender	Male	35 (62.5)	26 (51)	0.335
	Female	21 (37.5)	25(49)	
Mean age (years)		49.9 ± 8.1	48 ± 9.8	0.270
Hemoglobin (g/dl)		10.7 ± 1.7	11.5 ± 1.32	0.085
TLC (x10^9^/L)		6.8 ± 2.7	8.3 ± 2.2	0.003
Platelet (x10^9^/L)		122 ± 33	177 ± 39	≤ 0.001
Lymphocyte count		30.1 ± 7.3	27 ± 6.7	0.018
Neutrophil count		61.7 ± 7.9	64.4 ± 7.7	0.046
Serum creatinine (mg/dl)		0.8 ± 0.3	0.7 ± 0.3	0.014
Serum albumin (mg/dl)		2.9 ± 0.52	2.9 ± 0.66	0.494
Total bilirubin (mg/dl)		1.2 ± 0.55	1.1 ± 0.7	0.152
AST (IU/L)		48 ± 28	43 ± 22	0.351
ALT (IU/L)		42 ± 19	39 ± 17	0.427
GGT (IU/L)		106 ± 54	99 ± 45	0.516
INR		1.3 ± 0.27	1.1 ± 0.19	0.004
PLR		4.17 ± 1.44	6.8 ± 1.99	≤ 0.001
APRI		1.11 ± 0.8	0.65 ± 0.39	≤ 0.001
FIB-4		3.4 ± 2.2	2.1 ± 1.2	≤ 0.001

**Figure 1:**
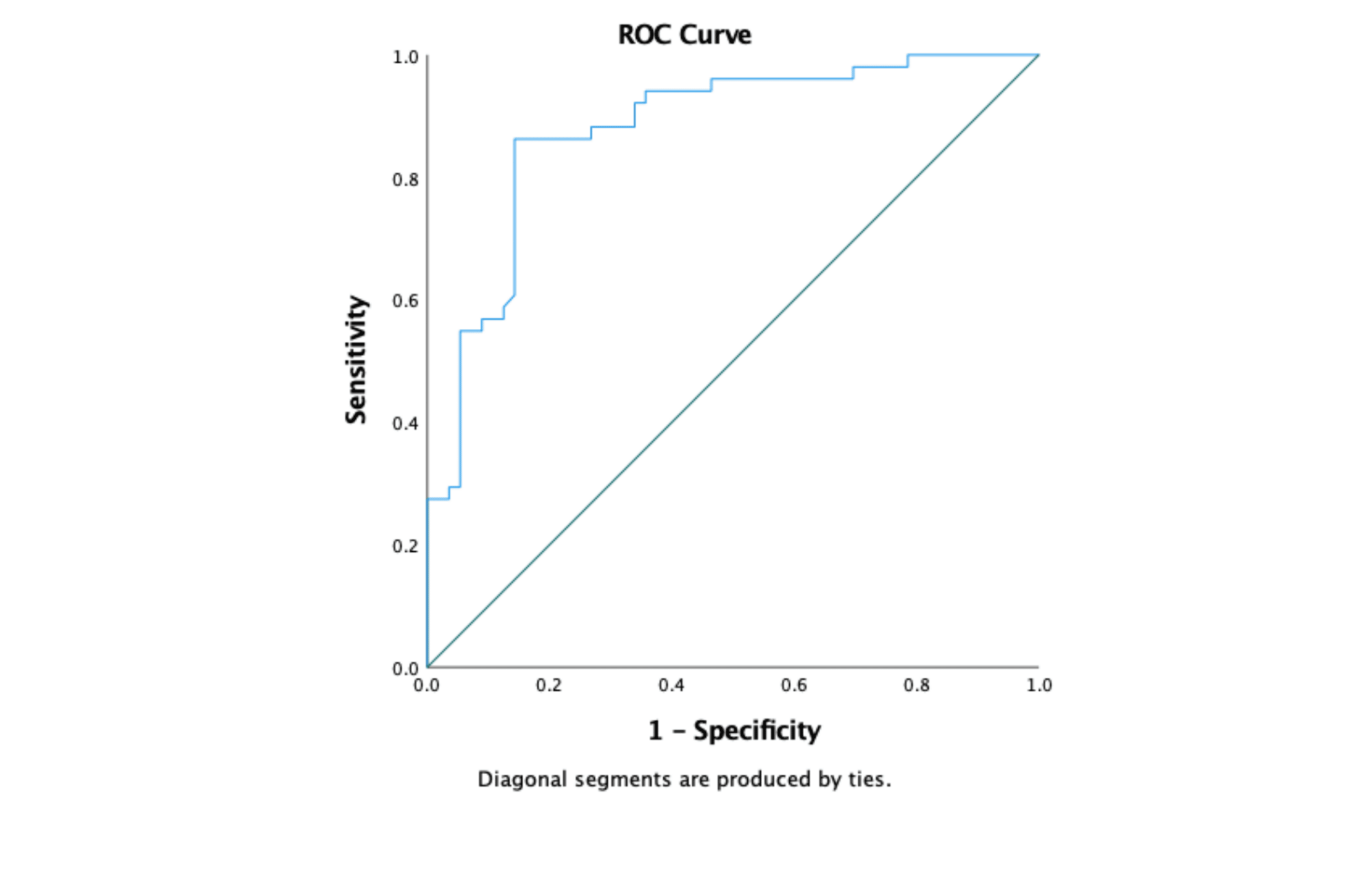
Area under the receiver operating curve (AUROC) for PLR in predicting advanced liver fibrosis is 0.879 (p ≤ 0.001) PLR: platelet-to-lymphocyte ratio; ROC: receiver operating curve

Area under the receiver operating characteristic (AUROC) for PLR in predicting advanced liver fibrosis was 0.879 (p ≤ 0.001) (Figure 1). PLR outperformed both APRI and FIB-4 in predicting advanced liver fibrosis. At an optimal cutoff of ≤5.41, PLR had a high sensitivity of 85.71% as well as a specificity of 86.27%, with a high diagnostic accuracy of 85.98%, making it a highly effective predictor of advanced fibrosis. APRI, in contrast, had poor sensitivity of 15.69% with a modest specificity of 57.14%, resulting in a poor overall diagnostic accuracy of 37.38%. Similarly, FIB-4 also had poor sensitivity of 23.53% with a specificity of 55.36%, with a corresponding overall accuracy of 40.19% (Table 3).

**Table 3:** Diagnostic accuracy of PLR, APRI, FIB-4, and SWE in predicting advanced liver fibrosis PLR: platelet-to-lymphocyte ratio; APRI: aspartate aminotransferase-to-platelet ratio index; FIB-4: fibrosis-4 index; SWE: shear-wave elastography; PPV: positive predictive value; NPV: negative predictive value

Variable	Sensitivity (%)	Specificity (%)	PPV (%)	NPV (%)	Diagnostic accuracy (%)
PLR	85.71	86.27	87.27	84.62	85.98
APRI	15.69	57.14	25	42.67	37.38
FIB-4	23.53	55.36	32.43	45.29	40.19
SWE	89.29	50.98	66.67	81.25	71.03

## Discussion

The outcome of this study indicates the efficacy of PLR as an accessible yet non-invasive biomarker to predict advanced liver fibrosis in patients with HCV infection. The findings of this study suggested that patients with advanced liver fibrosis had significantly low PLR with outstanding diagnostic performance indicated by an AUROC value of 0.879. Furthermore, this study also revealed that PLR was better compared to conventional non-invasive biomarkers such as the APRI and the FIB-4 in that it had higher sensitivity (85.71%) and specificity (86.27%). This suggests not only that PLR is an efficient screening tool but also that it can replace more expensive and invasive tests in resource-limited settings.

Previously, several studies have compared the role of non-invasive markers to predict advanced liver fibrosis in patients with HCV, including APRI and FIB-4 [9]. The current study emphasizes the predictive role of PLR in predicting advanced liver fibrosis in HCV infection by showing high diagnostic accuracy and overall performance.

Previously, Vallet-Pichard et al. proposed the usefulness of FIB-4 in predicting advanced liver fibrosis. However, it had limited accuracy in predicting intermediate stages of fibrosis [15]. In our study, FIB-4 showed an overall diagnostic accuracy of only 40.19% in determining advanced fibrosis in patients with HCV.

Correspondingly, Wai et al. (2003) have predicted the role of APRI as a non-invasive predictor with an AUROC value of 0.77 in determining significant fibrosis and an AUROC value of 0.89 in determining cirrhosis [16]. However, in our study, the diagnostic accuracy of APRI was considerably low (37.38%) as compared to APRI, FIB-4, and PLR, with a lower sensitivity in predicting advanced liver fibrosis. This can be explained by variation in population studied or methodology employed to detect fibrosis, but this suggests that PLR may be an improved tool in certain clinical contexts.

Previous studies have shown varied results regarding the role of PLR in predicting advanced liver fibrosis, particularly in patients with NAFLD [12]. However, the data is scarce regarding the role of PLR in predicting advanced liver fibrosis in HCV infection. Alsebaey et al. suggested the role of PLR in predicting both advanced liver fibrosis and insulin resistance in the HCV population [13]. These findings were similar to those observed in our study that establishes the role of PLR as an effective diagnostic marker. However, in our study, we noticed a higher diagnostic accuracy of PLR as compared to both APRI and FIB-4.

Several factors can be attributed to improved prediction by PLR over both APRI and FIB-4. APRI is predominantly reliant upon concentration of AST, which can independently change in response to non-fibrosing conditions such as acute inflammation or in response to abnormalities in metabolism [17]. FIB-4, including platelet count and age, is prone to bias by hematological status changes related to age [18]. However, PLR is derived by calculation from two stable hematological factors and is not biased by factors beyond these factors, providing an improved reflection of the development of fibrosis [13].

Despite its promising outcome, this study has certain limitations. First, because this is a retrospective work, this can induce selection bias. Second, while we have studied an appropriate cohort of patients with HCV infection, the sample number (n = 107) is relatively small in comparison to extensive multi-center trials. Third, antiviral therapy was not investigated to have an effect on PLR patterns in this work, an area to be studied in the future. These findings should be replicated in future, larger prospective cohorts.

## Conclusions

This study showed the significant utility of PLR as a non-invasive and cost-effective tool for the prediction of advanced liver fibrosis in patients with chronic HCV infection. PLR demonstrated superior diagnostic accuracy compared to APRI and FIB-4, with high sensitivity and specificity. Given its availability from routine CBC tests, PLR presents a reliable alternative for fibrosis assessment, particularly in resource-limited settings where access to liver biopsy and advanced imaging techniques is restricted. The ease of implementation of PLR in routine clinical practice underscores its potential as a valuable tool for early fibrosis detection and risk stratification in HCV-infected population. While the results of our study are promising, further validation in larger, prospective cohorts is necessary. Future research should explore combining PLR with other non-invasive biomarkers and imaging modalities to enhance diagnostic precision.
